# [Retracted] MicroRNA-302a targets GAB2 to suppress cell proliferation, migration and invasion of glioma

**DOI:** 10.3892/or.2025.9011

**Published:** 2025-10-27

**Authors:** Jiuhong Ma, Jia Yu, Jingmei Liu, Xing Yang, Miao Lou, Jinghui Liu, Fuqiang Feng, Peigang Ji, Liang Wang

Oncol Rep 37: 1159–1167, 2017; DOI: 10.3892/or.2016.5320

Following the publication of the above paper, it was drawn to the Editor's attention by a concerned reader that cell invasion and migration assay data featured in Fig. 5C on p. 1164 were strikingly similar to data that already been submitted for publication to the journal *Oncology Letters* in a paper written by different authors at different research institutes. Moreover, cell invasion and migration assay data panels both within Fig. 6C, and comparing Fig. 6C with Fig. 5C, revealed a number of internally overlapping panels, suggesting that data which were intended to have shown the results from differently performed experiments had apparently been derived from a smaller number of original sources.

Owing to the fact that the abovementioned data had already been submitted for publication prior to the receipt of this article at *Oncology Reports*, the Editor has decided that this paper should be retracted from the Journal. The authors were asked for an explanation to account for these concerns, but the Editorial Office did not receive a reply. The Editor apologizes to the readership for any inconvenience caused.

